# Basic principles of biobanking: from biological samples to precision medicine for patients

**DOI:** 10.1007/s00428-021-03151-0

**Published:** 2021-07-13

**Authors:** Laura Annaratone, Giuseppe De Palma, Giuseppina Bonizzi, Anna Sapino, Gerardo Botti, Enrico Berrino, Chiara Mannelli, Pamela Arcella, Simona Di Martino, Agostino Steffan, Maria Grazia Daidone, Vincenzo Canzonieri, Barbara Parodi, Angelo Virgilio Paradiso, Massimo Barberis, Caterina Marchiò, On behalf of Alleanza Contro il Cancro (ACC) Pathology and Biobanking Working Group

**Affiliations:** 1 Candiolo Cancer Institute, FPO-IRCCS, Candiolo, Italy; 2 Department of Medical Sciences, University of Turin, Turin, Italy; 3 Institutional BioBank, Experimental Oncology and Biobank Management Unit, IRCCS Istituto Tumori “Giovanni Paolo II”, Bari, Italy; 4 Unit of Histopathology and Molecular Diagnostics, Division of Pathology and Laboratory Medicine, IEO, European Institute of Oncology, IRCCS, Milan, Italy; 5 Istituto Nazionale Tumori, Fondazione G. Pascale, IRCCS, Naples, Italy; 6 Department of Oncology, University of Turin, Turin, Italy; 7 Department of Pathology, IRCCS Regina Elena National Cancer Institute, Rome, Italy; 8 Immunopathology and Cancer Biomarkers, IRCCS CRO Aviano-National Cancer Institute, Aviano, Italy; 9 Fondazione IRCCS Istituto Nazionale Dei Tumori, Milan, Italy; 10 Department of Medical, Surgical and Health Sciences, University of Trieste, Trieste, Italy; 11 Pathology Unit, IRCCS CRO Aviano-National Cancer Institute, Aviano, Italy; 12 IRCCS Ospedale Policlinico San Martino, Genoa, Italy

**Keywords:** Biobanking, Biospecimens, Tissue specimens, Cell lines, Standardization, Preanalytical phase

## Abstract

The term “biobanking” is often misapplied to any collection of human biological materials (biospecimens) regardless of requirements related to ethical and legal issues or the standardization of different processes involved in tissue collection. A proper definition of biobanks is large collections of biospecimens linked to relevant personal and health information (health records, family history, lifestyle, genetic information) that are held predominantly for use in health and medical research. In addition, the International Organization for Standardization, in illustrating the requirements for biobanking (ISO 20387:2018), stresses the concept of biobanks being legal entities driving the process of acquisition and storage together with some or all of the activities related to collection, preparation, preservation, testing, analysing and distributing defined biological material as well as related information and data. In this review article, we aim to discuss the basic principles of biobanking, spanning from definitions to classification systems, standardization processes and documents, sustainability and ethical and legal requirements. We also deal with emerging specimens that are currently being generated and shaping the so-called next-generation biobanking, and we provide pragmatic examples of cancer-associated biobanking by discussing the process behind the construction of a biobank and the infrastructures supporting the implementation of biobanking in scientific research.

## Introduction

*Time* magazine featured biobanks among “10 Ideas Changing the World Right Now” back in 2009 [1], highlighting biobanks as an opportunity for scientists and scientists alike to derive knowledge from thousands of samples. Starting from cancer, biobanks were linked to the ambitious chance of screening and treating any disease [1]. During the last decade, the field of biobanking has rapidly grown in parallel with the advent of precision medicine. The crucial role of biobanking research in personalized medicine has also been discussed by *Forbes* [2] in an article referring to the current evolution of biobanks from the usual collection of tissues and blood, nucleic acid, microbiome samples and stem cells to virtual biobanks, which raises the question of whether there is adequate infrastructural and economic support to foster the continuous rapid advance of biobanking. The International Agency for Research on Cancer (IARC) states that biobanks currently represent the foundation of three rapidly expanding domains of biomedical science: (i) molecular and genetic epidemiology (aimed at assessing the genetic and environmental basis of cancer causation in the general population as well as in families), (ii) molecular pathology (aimed at developing molecular-based classification and diagnostic procedures for cancers) and (iii) pharmacogenomics/pharmacoproteomics (aimed at understanding the correlation between an individual patient’s genotype or phenotype and response to drug treatment) [3].

## Biobanks: definition(s) and key features

Although the term “biobank” first appeared in scientific publications in 1996 [4, 5], there is still no agreement on a precise definition. The term “biobank” has been gradually adopted to describe any collections of biospecimens or human genetic data suitable for research purposes [6]. One of the first definitions, i.e. “a collection of biological material and the associated data and information stored in an organized system, for a population or a large subset of a population”, was introduced by the Organization for Economic Cooperation and Development (OECD) [5, 7]. This description was later updated to depict biobanks as “structured resources that can be used for the purpose of genetic research and which include (a) human biological materials and/or information generated from the analysis of the same and (b) extensive associated information” [8].

**Fig. 1:**
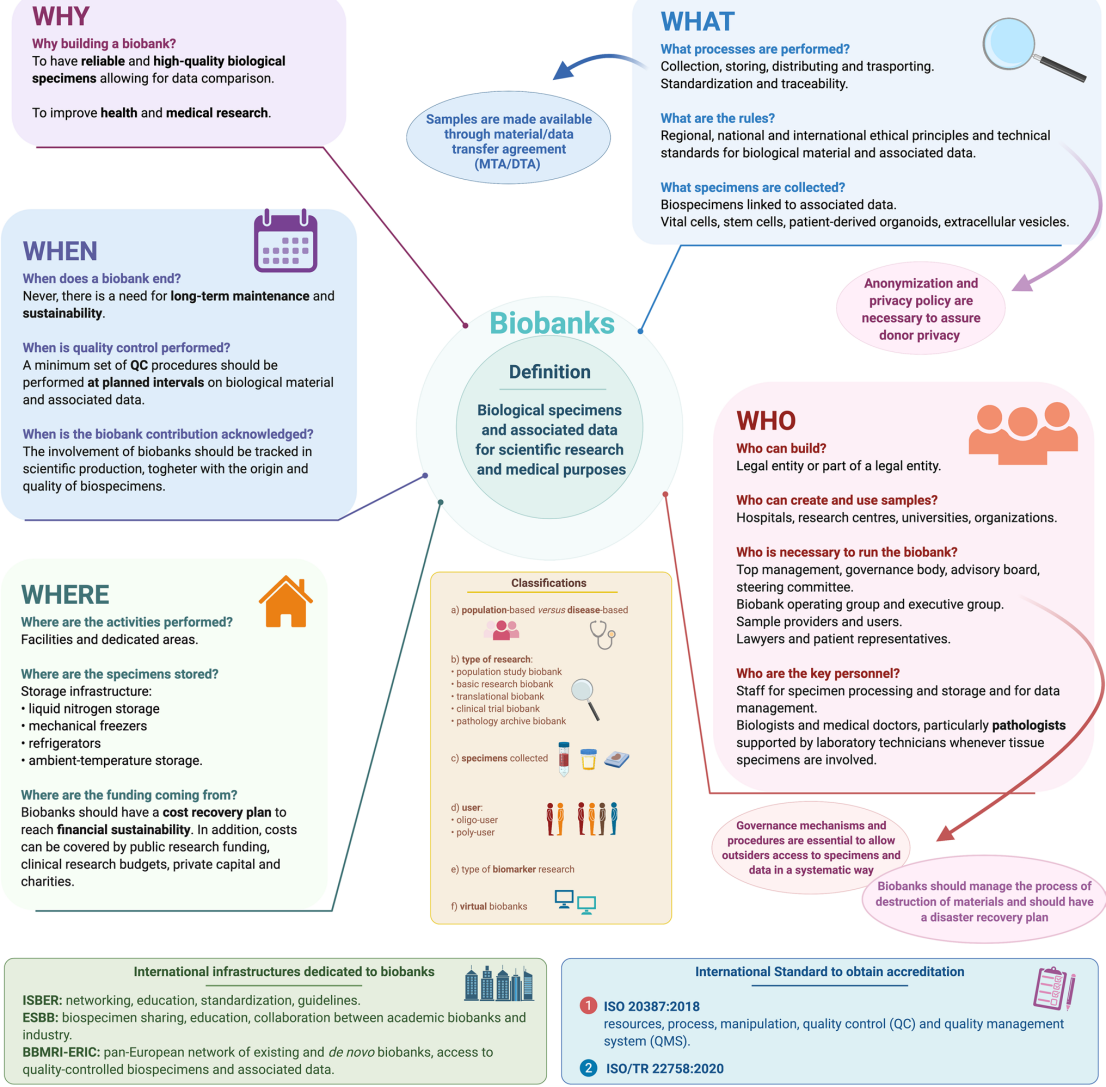
Basic principles of biobanking. Information is organized by exploiting the 5Ws’ approach (why, what, who, where and when), emphasizing definition, classification systems, key aspects, international standards required for accreditation and infrastructures needed to ensure quality and networking. (This image was created with BioRender: https://biorender.com/)

One unavoidable feature of biobanking is *the coexistence of biological specimens and associated data*. Biobanks are large collections of human biological materials linked to relevant personal and health information, which may include health records, family history, lifestyle and genetic information that are held predominantly for use in health and medical research [6, 9] (Fig. 1). Interestingly, the document produced by the International Organization for Standardization (ISO) illustrating the general requirements for biobanking (ISO 20387:2018) defines biobanks as legal entities or parts of a legal entity that perform biobanking and states that biobanking is the process of acquisitioning and storing, together with some or all of the activities related to collection, preparation and preservation and testing, analysing and distributing defined biological material as well as related information and data [10].

To accommodate advances in biotechnology and life science, the concept of biological resource centres (BRCs), infrastructures consisting of service providers and repositories of living cells, genomes of organisms and information relating to heredity and the functions of biological systems, was introduced by OECD [11]. Based on these definitions, boundaries between biobanks and other research collections cannot be considered clear-cut [6]. However, the European Commission highlights that biobanks are devoted to collecting biological samples and associated data for medical scientific research and diagnostic purposes and to organizing these in a systematic way [12]. In addition, the key factor that distinguishes a biobank from any other type of research collection is that *established* *governance mechanisms* *are in place* to allow outsiders access to resources in a systematic way [12–14].

Both biorepositories (ISBER 2001) and BRCs (OECD 2007) can include tissues from humans and animals as well as cell and bacterial cultures and even environmental samples. In contrast, a biobank typically handles human biospecimens and information about donors, such as demographic and lifestyle information, history of illness, treatment and clinical outcomes.

Since biobanks may have different backgrounds and aims, it is difficult to precisely list the *distinctive features of a given biobank*. Nevertheless, following the description provided by the European Commission biobanks:

(i) Typically collect and store biological materials that are annotated not only with medical but also often with epidemiological data (e.g. environmental exposures, lifestyle/occupational information)
(ii) Are not static “projects”, since biological materials and data are usually collected on a continuous or long-term basis
(iii) Are associated with current (defined) and/or future (not yet specified) research projects at the time of biospecimen collection
(iv) Apply coding or pseudonymization to assure donor privacy but have, under specific conditions, provisions that participants can be reidentified to provide clinically relevant information to the donor
(v) Include established governance structures and procedures that serve to protect donors’ rights and stakeholder interests [12]

In addition, biobanks are focused on the public interest rather than being concerned with individual benefits for the participants themselves and aim to provide a public benefit for future generations [6] through the translation and application of research findings [9].

It is important to acknowledge that biological samples are “pseudonymized” and not totally “anonymized”: this is key in being able to provide feedback to the owners of the samples, to recollect precious information and to reconnect them with the specimens.

## Classification systems

At present, no fully recognized guidelines for biobank classification are on record; nevertheless, a universal biobank classification system would be helpful to facilitate users in searching for biospecimens. Undoubtedly, biobanks are very heterogeneous, as they can differ in size, research topic, health status of the participants, specimens collected, procedures for sample collection and processing and storage systems [6, 12] (Fig. 1).

In the attempt to devise a classification, a first level of categorization acknowledges that biobanks can be “population-based” or “disease-oriented” (Fig. 1):

- *Population-based biobanks* provide specimens from individuals of a general population with the aim of studying the role of individual genetic susceptibility and exposure to external factors in the development of specific disorders by linking molecular data with other associated information [15].
- *Disease-oriented biobanks* collect disease-specific biospecimens. They may be focused on a single type of tissue or include biospecimens from different sources that are relevant to a disease such as cancer [15, 16].

Malsagova and colleagues reported that large-scale epidemiological research or collections from clinical trials of new medical interventions can represent a biobank [17]. Therefore, biobanks can be labelled *according to the type of research* they intend to support:

(i) Population study biobank
(ii) Basic research biobank
(iii) Translational study biobank
(iv) Clinical trial biobank
(v) Pathology archive biobank [18].

In addition, some have illustrated biobank categories based on the associated opportunities of biomarker discovery [19]:

(i) Population biobanks (biomarkers of individual genetic susceptibility and identity)
(ii) Disease-oriented and epidemiology-driven biobanks (biomarkers of exposure and biological effect)
(iii) Disease-specific biobanks, such as tumour banks [19].

A second method of classification considers *the type of samples collected*, such as biobanks collecting frozen tissues, formalin-fixed paraffin-embedded (FFPE) tissues, cells, whole blood and derivatives, urine, buccal cells and saliva, bone marrow aspirate, semen, hair, nails and nucleic acids (DNA, RNA, cDNA/mRNA, microRNA) [3, 15].

Watson and Barnes proposed a schema for classifying human research biobanks that was adopted by the Canadian Tumour Repository Network (CTRNet) [18, 20]. This system enables the categorization of biobanks following four functional elements: the type of donor/participant, the collection methods and design (e.g. retrospective or prospective accrual, size and scope), the features of the biospecimens (e.g. the predominant type of biospecimen preservation, such as fixed or frozen) and the nature of the brand and intended users (e.g. single group, institution or multiple users) [18, 20].

Finally, we should acknowledge a further category represented by virtual biobanks, i.e. electronic repositories of biological samples and other related data, regardless of where the real specimens are stored (Fig. 1) [16, 17, 21].

To have a practical idea of the biobanks available across the European area and of the type of samples they have at disposal, it may be useful to refer to the directory of the Biobanking and BioMolecular resources Research Infrastructure - European Research Infrastructure Consortium (BBMRI-ERIC) [22, 23]. In 2011, the catalogue included 63 population-based and 219 clinical biobanks, which in a few years has grown to 515 biobanks, representing more than 60 million biological samples [22, 24].

Biobanks can exist within hospitals, research centres, pharmaceutical companies and patient advocacy organizations. Biobanks located in an academic setting or as a part of a company reflect different cultures, aims and work practices characterized by a significant gap between them [25]. Academic biobanks are research-driven and usually supported by institutional funding and grants. In contrast, industry biobanks are more focused on end products and more business-oriented [25]. Despite these differences, there is a need for a reciprocal understanding of industry and academic backgrounds and to establish collaborations. For this to happen, it is necessary for industries to understand that human specimens and data cannot be treated as a commercial product and that biobanking is a scientific activity involving humans [25]. From the perspective of precision and personalized medicine, it is necessary that even biobanks start to move towards a patient-centred approach [26]. The Patient-Centered Outcomes Research Institute (PCORI) has established pathways for funding practical research by considering the patients’ interests [27, 28]. Patient-centred biobanking should look for ways to support investigators in conducting patient-centred research to make results more useful in healthcare decision-making [26, 28]. In the context of patient-centred biobanking, it is interesting to highlight the experience of the PATH Biobank (Patientsʼ Tumour Bank of Hope), a non-profit biobank in Germany founded by breast cancer survivors and dedicated to supporting breast cancer research, both in academic and industrial fields [29]. This approach highlights how the role of patients in biobanking is changing: from simple donors to an active part [26]. Interestingly, within PATH Biobank at the end of the diagnostic process, the leftover is divided into two parts, one remaining at the disposal of the patient and the other being dedicated to research. After the death of the patient, the patient’s sample becomes a property of PATH and is available for research [29].

## What to know when building a biobank

Given the complexity of biospecimen handling and management, setting up a biobank may be challenging (Fig. 1). Harati and colleagues tried to provide indications for the creation of a biobank, including accreditation, standards of practice and funding issues [16]. A guidance document produced by the government of South Australia suggests that a defined purpose or business plan is key, and governance, funding and other financial considerations, data and specimen management and consent must be considered [9]. In addition, the process of accreditation and the observation of the standards of practice allow biobanks to operate professionally and to provide biological specimens of adequate quality [17].

It is necessary to prioritize *ethics*, *privacy*, *informed consent*, *data security* and *standardization* (Fig. 1). According to the IARC, developing biobanks involves ethical, legal and social issues (ELSI) and requires the design of governance systems [3]. IARC’s recommendations are based on guidelines that incorporate the knowledge gained from projects such as Standardization and Improvement of Generic Preanalytical Tools and Procedures for In Vitro Diagnostics (SPIDIA), BBMRI – Large Prospective Cohorts (BBMRI-LPC) and the International Genomics Consortium (IGC) as well as the European Committee for Standardization (French, Comité Européen de Normalisation, CEN), Technical Specifications for molecular in vitro diagnostic examinations and International Organization for Standardization (ISO) norm [3].

According to the IARC, the following key features should be considered when creating a biobank:

- Type, number, aliquots, size of biospecimens
- Storage containers
- Storage temperature and conditions
- Frequency of access to biospecimens
- Requirements for identification of biospecimens
- Availability of storage space
- Requirements for temperature monitoring
- Associated data
- Financial and operational sustainability [3].

The IARC document also provides protocols for sample processing and useful templates for a consent form and for a *material/data transfer agreement* (MTA/DTA) (Fig. 1) [3, 30].

An important aspect of the creation, reliability and sustainability of a biobank is the standardization of processes connected with sampling, storage and *quality control* (QC). In recent years, specific projects on the standardization of preanalytical, analytical and postanalytical procedures in scientific laboratories, including biobanks, have been undertaken. For instance, the “SPIDIA” project was launched by the European Union FP7 programme in 2008, with the participation of leading academic institutions, international organizations and life sciences companies. The project specifically addressed the standardization and improvement of preanalytical procedures for in vitro diagnostics. Within CEN/Technical Committee 140 for “In vitro medical devices”, the SPIDIA results enabled the development and introduction of the first 9 CEN Technical Specifications (CEN/TS) for preanalytical workflows in Europe. In 2017, the SPIDIA4P project was built on the SPIDIA results to develop and implement a comprehensive portfolio of an additional 14 pan-European preanalytical CEN/TS and ISO/IS documents as well as external quality assessment schemes (EQAs), addressing the important preanalytical workflows for personalized medicine. SPIDIA4P was recently acknowledged as one of three success stories by the European Commission.

Information technology (IT), data systems and record administration are also critical aspects of biobanks, and efforts should be made to guarantee that these elements are effective and secure [16]. For excellent biobank implementation, it is important to have a good system for sample traceability, in particular exploiting a barcoding system and an IT platform integrated with all institutional operating systems to automatically integrate data, thus avoiding potential errors stemming from manual entry.

Biobanks shall *ensure* not only *traceability of biological material and associated data* but also destruction [10]. Indeed, biobanks should be able to *manage the process of destruction of biological material and/or deletion of associated data* beyond any possible reconstruction. A legacy plan should be formulated to guide who, what, when, where, why and how specimens and associated data should be transferred or destroyed following a specific event [31]. From an ethical perspective, destruction of samples after consent for use is not usually included in the informed consent: biobanks traditionally communicate to participants how the samples will be used to support biomedical research initiatives, and this implies that the material will be used in the future. Biobanks may include appropriate disclosures about the potential destruction of specimens in their statements or consent documents, considering that representing and safeguarding the interests of those who have donated samples are a full responsibility of a given biobank [31].

Finally, biobanks should *define disaster recovery plans* to avoid loss of biological material following natural and human-made disasters [3, 10, 32]. The IARC document provides detailed guidance on how to define the recovery plan and what are the key steps to be considered. These include categorizing samples in order of priority, listing actions to be performed through standard operating procedures (SOPs) and ensuring adequate backup storage in case samples need to be transferred [3].

## Financial and operational sustainability of biobanks

Biobanks need funding for development, staff management and long-term maintenance [16]. As recently reported by the Biobanking Financial Sustainability survey of the National Cancer Institute’s Biorepositories and Biospecimen Research Branch, the majority of biobanks do not have plans for long-term sustainability, rather they are supported by public research funding; they are often not autonomous from their host organizations, which are also usually dependent on publicly funded research programs [33].

Although biobanks have explored several funding models - extramural and intramural funding from private capital, government funds and charities - *ensuring long-term sustainability is difficult*. Data on cost recovery are not promising: many biobanks recover insignificant amounts of fees in relation to their operational costs [34]. An international group of experts has set a tool to assign prices to access specimens and data [35]. The instrument was tested by 16 European biobanks, and this experience demonstrated that financial sustainability can only be achieved if the biobank applies a cost recovery policy based on user fees that reflect the true costs faced by biobanks [35]. A valuable economic model should consider the needs of the market and the key processes of biobanking, i.e. the costs of case collection, tissue processing, storage management, sample distribution, infrastructure and administration [36, 37]. A model that takes these factors into account, such as the total life cycle cost of ownership (TLCO) model, allows for a better definition of the variable and fixed costs of the biobank, which are necessary to implement cost recovery programs [36].

There is not yet a prevailing or accepted procedure to reach financial sustainability; however combining traditional ways to new approaches to build novel sustainability and business models that respond to specific requirements of biobanks may be key. Improving the value to society and public benefit, addressing the interests of funders, researchers and participants, may help enhance the value of biobank resources and improve their long-term sustainability [34]. As suggested by Simeon-Dubach and Watson with the concept of biobanking 3.0, the key to achieving economic sustainability lies in the ability to improve the different stakeholders’ perceptions of the biobank [38].

Biobanks have also to address *the problem of underuse of biospecimens and data*, which can significantly impact sustainability. Not all biobanks represent success stories, such as the tissue bank of the National Center of Tumor Diseases in Heidelberg (NCT), which has been able to provide high-quality tissues for 605 research projects in less than 6 years [39]. Access to quality tissue specimens, together with high numbers of projects involving biobanking, high project completion rates and high user satisfaction rates, are instrumental to success [39]. Measuring biobank outputs can provide all stakeholders with reliable data on the value of the biobank, which in turn may help increase usage, better address research needs and alleviate some risks to biobank sustainability [40]. However, quantitative data about the usage and the contribution of biobanks to research are still hard to retrieve [41], and it is not easy to understand whether the investment in the biobank provides a good payback for science. This is, for example, the case of the EFS Centre-Atlantique donor’s biobank that, after 10 years of sample usage, experienced underutilization of the available resources [42]. The main causes of underusage can be the low or undocumented quality of samples, an inefficient model of governance, restricted policies that prevent the involvement of biobanks in translational research, a lack of proper advertisement of available collections and the limited involvement of patients and civil society in direct governance of biobanks [43].

## International infrastructures dedicated to biobanks

The role of infrastructures in biobanking is to arrange encounters among researchers, biobanks, industry and patients to offer tools, software, quality management services and support with ethical and legal issues.

Major international infrastructures dedicated to biobanks are summarized here below:

- The International Society for Biological and Environmental Repositories (ISBER) was established in 1999 [44], with the mission to facilitate and promote networking, to encourage education and improvements and to standardize approaches for biobanking. One of its main aims is to produce guidelines to guarantee high-quality specimens for future research. The “ISBER Best Practices: Recommendations for Repositories” reports the most successful procedures for sample management, including either evidence-based or consensus-based practices for the collection, long-term storage, retrieval and distribution of specimens [45]. These guidelines are periodically improved to reflect progress in research and technology [44].
- The European, Middle Eastern and African Society for Biopreservation and Biobanking (ESBB) was founded in 2010 [46]. Its mission is to improve biospecimen sharing by encouraging and educating the biobank community. It promotes collaborations between biobankers, enhancing cooperation between academic biobanks and industry [46].
- In 2006, the European Strategy Forum on Research Infrastructures (ESFRI) listed in its roadmap “a pan-European and broadly accessible network of existing and de novo biobanks and biomolecular resources” [47]. On that occasion, the Biobanking and BioMolecular Resource Research Infrastructure (BBMRI) was proposed [47, 48]. From 2008 to 2011, BBMRI was granted funding by the European Commission through the European Framework Programme 7 [49], and in 2013, it was officially awarded the community legal framework for a European Research Infrastructure Consortium (ERIC) [50, 51]. BBMRI-ERIC aims to provide access to quality-controlled biospecimens and associated data for cross-biobanking research [3, 51]. It currently consists of 21 European member states, international organizations and observers. The role of BBMRI-ERIC is to manage the directory of European biobanks and to offer support to biobanks in terms of quality management, information technology, ELSI and General Data Protection Regulation (GDPR). The BBMRI-ERIC carries out its actions through the active participation of national nodes.

## A focus on cancer-oriented biobanks

Cancer-oriented biobanks aim to collect and store human biological samples for cancer research. To date, cancer-oriented biobanks are based on the collection of biological samples from patients with a specific disease (cancer) and controls, i.e. healthy tissues from cancer patients, and represent a long-term source of human biological samples with associated information, collected at the time of diagnosis and during consecutive therapeutic phases (e.g. before and during therapy, at follow-up and in case of relapse).

**Fig. 2:**
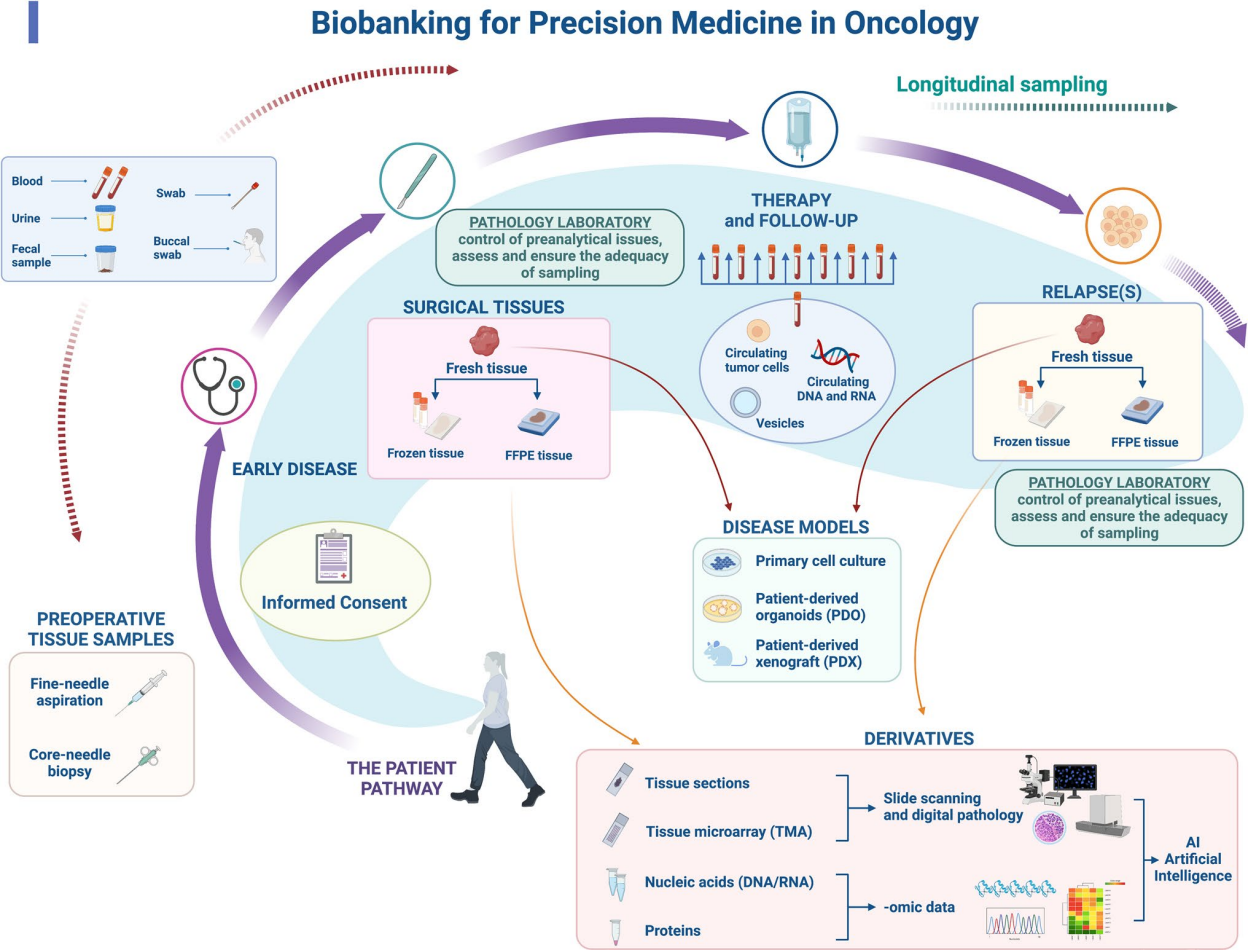
Biobanking for precision medicine in oncology. Practical example of the potential of biobanking for precision medicine in oncology. The pathway of a given patient is illustrated depicting the possible contribution of biobanking in the patient’s clinical history, either in early or advanced disease stage. Provided that informed consent is signed by the patient, different biological specimens may be collected. In the preoperative phase, samples can be obtained through fine-needle aspiration and core-needle biopsy. The same applies to metastatic lesions that are typically investigated to confirm the origin of the disease and to perform biomarker analyses for additional therapeutic strategies. In early-stage disease, patients undergo surgical resection. In each step, the pathologists play a key role in managing the preanalytical phase and ensuring that sampling is appropriate and does not impact the final diagnosis. Specimens (in particular surgical specimens) can be freshly sampled and used for the preparation of disease models (such as primary cell culture, PDO and PDX). Tissues can also be snap frozen and are always formalin-fixed and paraffin-embedded (FFPE) for diagnostic purposes. From tissue sections, proteins and nucleic acids for high-throughput genomic analyses can be obtained. Finally, tissue sections can now be systematically digitally scanned to foster the creation of a digital pathology archive. This wealth of tissue-related data stemming from different analyses can be exploited for the development of artificial intelligence (AI) approaches. Blood, urine, faecal samples and swabs can also be collected throughout the course of the disease. Longitudinal sampling may be performed during therapy and over the follow-up period. Circulating tumour cells, nucleic acids and vesicles can be isolated from blood samples (liquid biopsy). (This image was created with BioRender: https://biorender.com/)

Since tissue samples are key elements in most of the cases in these biobanks, an important role is played by *pathology laboratories* (Fig. 1, Fig. 2), which (i) handle specimens, (ii) assess and ensure the adequacy of fresh sampling and (iii) represent the tissue curators and are responsible for FFPE specimen archives. In addition, *clinical pathology laboratories* are involved in the collection of whole blood and derivatives for routine purposes: this is important since liquid biopsies are collected in different scenarios, including clinical trials and translational studies [52, 53] (Fig. 2).

Pathology laboratories may represent the connection between samples and biobanks, or they could be part of a given biobank; nevertheless, strict collaboration with pathology laboratories is necessary in light of the control of *preanalytical issues*, such as cold ischaemia time and time to fixation (the latter for FFPE tissue specimens), which are essential to guarantee the quality of tissue samples and their derivatives for molecular (high-throughput) analyses. In this respect, for FFPE tissue samples, the time of fixation is also important since formalin fixation impacts DNA/RNA fragmentation and therefore affects the success of downstream molecular analyses. Recently, the temperature of formalin fixation has been shown to matter [54, 55], and cold fixation can be considered when aiming to obtain a lesser degree of nucleic acid degradation [54–58].

Of note, tissues sampled for research purposes and therefore stored in a biobank may stem from “leftover tissues” from surgical specimens or derive from minor surgery, endoscopy or ultrasound-guided biopsies. Tissue samples are always obtained by preserving the diagnostic process, which has priority over the collection of the biobank, and conversely, any sample stored in the biobank is available for diagnostic integration. SOPs should be well defined and strictly followed: samples are typically collected within 15–20 min after surgery and immediately frozen with or without inclusion in optimal cutting temperature compound (OCT) to avoid drying artefacts, prolonged exposure to room temperature, cold ischaemia and crushing artefacts due to the procedure. The standardization process includes some QC schemes on aliquots the surgical specimen for histopathological and molecular checks during the sampling phase of fresh tissues or in case of withdrawal from frozen and stored samples. In pathology laboratories and biobank facilities, the possibility of optimizing access to the samples by aliquoting the tissues embedded in OCT and by performing cryostatic sections and making the frozen sections instead of the entire block available can be offered. In selected cases, macro- and microdissection procedures are also used.

Taken together, each step here described in the *specimen workflow is key to guarantee the success of precision medicine*, given that the quality of the sample is the fundamental prerequisite for any reliable data analysis, stemming from either single biomarker investigations or high-throughput analyses.

## Next-generation biobanking aiming to increase precision in medicine

Interest in biobanking activity has recently been addressed to new products and procedures required by new research approaches. Biological samples that are stored for research purposes are indeed changing: one example is vital cells (e.g. blood, bone marrow, foetal tissue, umbilical cord cells and fertilized eggs) or stem cell lines. Many benefits can be obtained by stem cell banking; however, standardization and QC during banking procedures are mandatory to allow scientists and scientists alike to evaluate their results and to develop safe and effective new therapies [59].

Another example is offered by the large numbers of different patient-derived xenograft (PDX) and patient-derived organoid (PDO) cultures that are being created, which represent a significant resource of living biobanks (Fig. 2) [60–64]. These samples constitute relevant preclinical cancer models, and they are instrumental for translational research aimed at confirming the therapeutic efficacy of a compound or discovering new therapeutic approaches [64]. Indeed, it has been shown that these samples retain key properties of native tumours and are therefore useful for revealing patient-specific drug sensitivities by drug screening at the service of precision medicine [61, 62, 65–68].

PDXs and PDOs are stored for an extended period of time that can be virtually unlimited. In addition, they are very time-consuming and consequently very expensive. In this respect, we should acknowledge that some international collaborative efforts focused on the use of PDX models have been established, such as the NCI’s PDXNet and the EurOPDX Consortium [69]. As an example, the essence of EurOPDX stems from two main needs: (i) mutualising efforts, thus exchanging models and expertise to avoid duplication, and (ii) raising standards in preclinical cancer research to significantly improve the success of drug development in the field of oncology [70].

When working with PDXs and PDOs, it may also be useful to know about the activity of The Living Biobank at the Princess Margaret (PMLB) Cancer Centre [71], which is a collaboration between PM researchers and the UHN Biospecimen Core to establish a central repository and provide services for the use of PDO and PDX models. For instance, PMLB generates a variety of tumour and normal organoid models of the intestine, pancreas, lung, mammary gland and oesophagus. Each organoid model was extensively characterized (STR matched to patient tissue, histopathology, mycoplasma testing, doubling rate information), and organoid models were accessible to internal/external researchers.

Central to the creation of PDXs and PDOs is the viability of tissues that are sampled from excised organs. It has been shown that an increase in surgical time strongly reduces cell viability, and both warm ischaemia and cold ischaemia times may affect cell viability and inversely correlate with the engraftment rate [72, 73]. If we cannot impact the duration of surgical resection on one side, it is important to maintain cold ischaemia time as short as possible. The implementation of new procedures for specimen transportation and storage from surgical theatres to pathology laboratories, such as the use of under-vacuum technology, enables fresh sampling in circumstances in which this would be troublesome [74]. Under-vacuum sealing per se is not entirely effective and shall always be associated with a controlled temperature of 4 °C to be maintained until sampling [72]. In addition, according to our experience, whenever successful generation of PDOs is suboptimal immediate fresh sampling (< 30 min from excision without vacuum sealing of the specimen) should be favoured. These data highlight once more the precious contribution a correct specimen handling by pathologists can offer to precision medicine.

*Extracellular vesicles* can be even harder to collect and store: to be effective, this specific biobanking practice should be based on fully optimized and standardized collection, isolation and storage protocols as well as on the use of universal markers that completely characterize the extracellular vesicles [75].

Another example of increasing importance is offered by the field of *metabolomics* or *lipidomics*. Indeed, metabolomics and lipidomics can provide useful data on disease evolution and prognosis or on reactions to nutrition or drug compounds [76]. Since the concentration of metabolites can be affected by several factors, i.e. ongoing enzymatic activities, temperature and oxygen exposure, standardized and validated protocols must be used for robust metabolomics and lipidomics tests [77]. It is important to note that some metabolite or bioactive lipid analyses require tight collection and storage procedures, hence the analysis may be difficult from biobanked tissues [76]. For this reason, it may be necessary to schedule an *ad hoc* collection for samples to be subjected to this type of analysis. In addition, multiple representative aliquots should be obtained, when possible, because multiple freeze–thaw cycles may be detrimental to metabolites [76, 77].

Other novel important aspects that are currently being evaluated in the scenario of biobanking tending to precision medicine are related to the concept of monitoring natural disease history and enriching clinical data associated with biological samples. Multiple samples from the same patient and tissues collected from patients at different time points in specified clinical contexts are strategic for biomarker investigation and discovery using multidimensional high-throughput technologies [78, 79]. *Sequential sampling* enables detailed studies of tumour evolution/progression and provides specimens for creating new cell lines and patient-derived xenografts for translational research (Fig. 2). To reach this goal, sampling should be standardized, and specimens must be fully annotated [80].

In contrast to classic biobanking, next-generation biospecimens are therefore likely to be collected more frequently within the context of therapeutic trials, with specific requirements and costs likely to also be covered by clinical research budgets.

## Biobanking as a matter of scientific productivity and transparency in scientific research

The bioresource research impact factor/framework (BRIF) initiative has addressed the issue of the value of biobanks in terms of research impact [81]. Its main aim is to develop a quantitative tool to *recognize and measure the use and impact of biological resources in health research*. BRIF systematically tracks and quantifies the use of a bioresource in the academic literature, thus enabling recognition of the work performed and encouraging stakeholders to efficiently share these resources [81, 82]. Keeping track of the use of a bioresource is the first step in this process, and new tools have been developed or are being developed to make this goal feasible: the CoBRA (Citation of BioResources in journal Articles) guide, the Open Journal of Bioresources (OJB) and the BRIF metrics [82]. Ideally, a unique digital identifier assigned through existing mechanisms (along the lines of DOI) should be used to cite and acknowledge the use of bioresources in publications and research projects.

In addition to tracking the involvement of biobanks in scientific production, it would also be advisable to be able to *verify the origin and quality of specimens used in scientific papers*. A lack of quality control is damaging the scientific literature by spreading misinformation [83]. Since preanalytics and processing methods may impact the sample quality, some have proposed an approach to encourage transparency and improve reproducibility in science by suggesting biorepositories to deposit their SOPs in a centralized database. The database enables linking SOPs to the sample collections and assigns an ID to the sample preparation SOP that can be reported in the methods section. This would allow scientists to obtain details on how the samples were processed, to find suitable collections for their experiments and to know the details of the treatment to compare the results obtained with samples from different biobanks [84].

## Biobanking at the time of the COVID-19 pandemic

The outbreak early in 2020 of the novel coronavirus, also known as severe acute respiratory syndrome coronavirus 2 (SARS-CoV-2), led to a global pandemic of COVID-19. As recently highlighted in an editorial on the possible consequences of the COVID-19 pandemic for the use of biospecimens from cancer biobanks, the risks associated with the collection and processing of human biospecimens with unknown status in relation to SARS-CoV-2, whether for diagnostic, therapeutic or research purposes, should be considered [85].

In this context, BBMRI-ERIC has produced a document to provide a list of resources that researchers working against COVID-19 can access via the BBMRI-ERIC network [86, 87]. Biobanks aim to support those who are working on COVID-19; however, adherence to best practices for the safe handling, processing, storage and shipment of biospecimens to reduce health risks to biobank staff is mandatory and of the utmost importance [88]. BBMRI-ERIC has included an important disclaimer in its document that samples should be collected only by biobanks that are properly equipped [87]. Biobanks should strictly observe the WHO laboratory *biosafety guidance related to COVID-19* [89]. The Centers for Disease Control and Prevention (CDC) of the USA have published guidelines for managing COVID-19 specimens [90]. Some biobanks, such as the University of California San Francisco, have established their own guidelines [91]. Overall, these recommendations dictate the use of biosafety level 2 rules for laboratories and appropriate disinfection practices [92].

Finally, considering that all types of biospecimens and organs are potentially affected by COVID-19, these precautions should be applied to all samples. A possible way to prevent consequences of the SARS-CoV-2 pandemic on the future use of materials from biobanks for research activities is for biobanks to separately store all human samples collected during the COVID-19 outbreak [85].

Undoubtedly, the COVID-19 pandemic has led to a new, critical and urgent challenge in the field of biobanking and has highlighted the power of biobanking as well as the need for accurate quality assurance, traceability and financial investment in biobanking to allow scientific research to be conducted while guaranteeing the safety of all procedures and personnel.

## International Standard ISO 20387:2018: “Biotechnology - Biobanking - General requirements for biobanking”

The ISO is an independent, nongovernmental international organization in which 165 national standards bodies participate, with one member per country. Through the action of experts, the ISO aims to share knowledge and produce consensus-based international standards that support innovation and deliver solutions to global challenges [93]. International standards represent documents that offer guidance, practical information and best practices created by people who will use and be impacted by them, so-called experts (Fig. 1). ISO standards comprise therefore rules, guidelines, processes, specifications or characteristics to standardize procedures and allow users to perform tasks in consistent and repeatable ways. Standards set minimum requirements for safety, reliability, efficiency and trust. In addition, the ISO ensures that these requirements are accepted in all member countries [94]. In 2018, the first ISO document (ISO 20387:2018) aiming to define the general requirements for the competence, impartiality and consistent operation of biobanks was released [10] (Table 1). This document is addressed to all organizations performing biobanking for research and development (it does not apply to clinical and therapeutic diagnostic biobank), and it has been an important milestone for the harmonization of procedures at international level [15]. ISO 20387:2018 allows biobanks to obtain accreditation for their activities, thus formalizing their competence [95].

**Table 1:** International Standard ISO for biobanking: scopes and structure

ISO document	Scope	Main features discussed within the document	Key points (“take home messages”)
ISO 20387:2018	To define the general requirements for the competence, impartiality and consistent operation of biobanks, including quality control requirements	• General requirements • Structural requirements • Resource requirements • Process requirements • Quality management system requirements	• The requirements defined by ISO 20387:2018 concern resources, processes, manipulation, QC of biological material and associated data and the quality management system • The document contains a “Confidentiality” section stating that biobanks should protect the confidential information and proprietary rights of providers/donors, recipients and users • A biobank should have procedures for safe handling, packaging, transport and reception relevant to the biological material concerned and should ensure the traceability of biological material and associated data from collection and acquisition or reception to distribution and disposal or destruction • The specimen collection procedure should follow workflows based on existing ISO documents • ISO 20387:2018 emphasizes that when the material for the biobank coincides with samples requiring clinical assessment and/or diagnosis, the process should be performed by qualified personnel and that specimen collection should never affect patient care • The reliability of the collected specimens is assured by QC activities that biobanks should perform to demonstrate the fitness for the intended purpose of the biological material and associated data and to provide objective evidence of the comparability and quality of biological material
ISO/TR 22758:2020	• To provide support in the implementation of the requirements by ISO 20,387:2018 • To achieve staff expertise and the proper quality of biological material and data collections	• Conformity with ISO 20387:2018 • Interpretation of ISO 20387:2018 requirements	

Legend: *ISO*, International Organization for Standardization; *QC*, quality control

A new document has been published, ISO/TR 22758:2020 “Biotechnology - Biobanking - Implementation guide for ISO 20,387”, which provides support for implementing the requirements of ISO 20387:2018 [96] (Table 1)

## Conclusions

Biobanks represent a fundamental organ to foster scientific research by guaranteeing the quality of results and adherence to standard laboratory practices and ethical requirements. The delicate functioning of biobanks requires governance, organization (at scientific, technical and administrative levels) and specific funding. Many actors play roles in this process, and the integration of different expertise is key. When addressing tissue-based research, particularly with cancer tissues, collaboration with pathology laboratories that curate tissue samples is of the utmost importance to accurately manage specimens starting from the preanalytical phase to the analytic process, which can be directed to tumour cells, the microenvironment or both. In this field, next-generation biobanking is rapidly emerging, featuring the generation of organoids as “avatars” of different neoplastic lesions that can be instrumental to answer open questions in translational research.

Biobanks demand safety, reliability, efficiency and trust. To fulfil these requirements, ISO standards, which are documents that comprise rules, guidelines, processes, specifications or characteristics to standardize procedures and allow users to perform tasks in consistent and repeatable ways, are available. Finally, when operating in biobanks, participation in dedicated international infrastructures is advisable, and this can facilitate networking, encourage education, improve standardization and support recognition of biobanks as a vital part of scientific productivity.

## Data Availability

Not applicable.
